# Sensors in Collaboration Increase Individual Potentialities

**DOI:** 10.3390/s120404892

**Published:** 2012-04-13

**Authors:** Gonzalo Pajares

**Affiliations:** Department of Software Engineering and Artificial Intelligence, Faculty of Informatics, University Complutense of Madrid, 28040 Madrid, Spain; E-Mail: pajares@fdi.ucm.es; Tel.: +34-1-394-7546; Fax: +34-1-394-7547

Different applications require different sensor technologies and methods to achieve specific goals. Particular sensor designs are focused on solving problems. It is well-known that individual sensors can be limited when complex problems or applications are involved or the application requires sensing in different locations or even different geographical areas.

We could think of robotic applications where vision, ultrasounds or tactile technologies among others are considered as a whole with the goal of navigation and exploration. Individual sensors are insufficient for achieving the goal, but in collaboration the objective can be achieved and even with high effectiveness. Some sensor devices are arrays of single elements, such as tactile or electronic-noses. In both cases, sensors are related to a specific location. On the contrary, some applications are based on the distribution of sensors at different locations, interconnected under a network for collaboration. At each specific location different sensors can be working in collaboration.

Works on this special issue cover the topic of collaborative sensors under different configurations, always enhancing and improving performances of individual sensors, based on the fusion of the information provided by the different sensors. Different categories and specific applications are considered, where each work is assigned to one or more specific categories and listed in the appropriate references section.

## *Wireless Sensor Networks* (WSN)

(1) coverage precedence routing algorithm, ensuring full functionality, for quality of service in WSN, [1]; (2) Diffusion-based Expectation-Maximization algorithm for energy-efficient solution in WSN [2]; (3) trust Index based Subtract on Negative Add on Positive (TISNAP) localization algorithm for multiple event source localization using binary information from the sensor nodes in WSN [3]; (4) collaborative localization algorithms for nodes in WSN without GPS [4]; (5) prediction (data not sent to the sink node) accuracy for data reduction in WSN [5]; (6) grid-based distributed event detection scheme for WSN [6]; (7) WSNs for intelligent transportation systems [7]; remote testbed with WSN and mobile robots equipped with a set of low-cost off-the-shelf sensors for cooperative perception [8]; (8) wireless body area networks for monitoring health parameters are useful for transmitting data externally [9]; (9) distributed and formula-based bilateration algorithm used to provide initial set of locations in WSN [10]; (10) Artificial neural network to estimate the location of a mobile station in wireless communication systems [11]; (11) WSN and minimax method in early detection to neutralize intruders in strategic installations [12].

## Medicine and Health Services

(1) wireless wearable and ambient sensors that cooperate to monitor person's vital signs such as heart rate and blood pressure during daily activities [13]; (2) body sensor networks with wireless technology can be used for the acquisition of health related information, which is transmitted to an external gateway, such as a PDA [14]

## Inertial Measurement Units

(1) fusion algorithms for using multiple Inertial Measurement Unit (IMUs) to enhance performance in the context of pedestrian navigation [14]; (2) a set of distributed accelerometers are arranged and integrated as an IMU [15].

## *Micro-Electro-Mechanical Systems* (MEMS)

(1) based on low-cost sensors along buried pipes in communication with a smart server for decision making [16]; (2) body sensor networks for health purposes [9].

## Security in Intelligent Sensors

patterns-based security specifications and new ontological specification [17].

## Oceanographic and Meteorological

instruments are installed on a buoy as a multisensory moored platform for continuous and autonomous monitoring of the pelagic system in Western Mediterranean [18].

## Robotics

(1) Odometry and laser scanners are integrated for relative localization for navigation of a convoy of robotic units in indoor environments [19]; (2) autonomous robot-arm model for object manipulation in semi-structured environments based on an intelligent multi-sensor system [20]; (3) specific tasks are distributed and allocated to each element in a swarm robotics by applying optimization methods, such as genetic algorithms [21]; (4) social odometry, where robots learn from the others, based on cooperative reputation systems [22]; (5) 3D parallel mechanism robot-arm with three pneumatic actuators combined with a stereo vision system is developed for path tracking control [23]; (6) remote testbed with mobile robots and WSN equipped with a set of low-cost off-the-shelf sensors in cooperative perception, that present high degree of heterogeneity in their technology, sensed magnitudes, features, output bandwidth, interfaces and power consumption [8].

## Automatic House

heterogeneous collaborative sensor networks for electrical and energy management on a self-sufficient solar house [24].

## Gyroscope

a design of force to rebalance control for a hemispherical resonator gyro (HRG) based on FPGA [25].

## 3D Structure

(1) fusion of stereovision and range finder sensors applied to autonomous vehicles guidance [26];(2) fusion of Kinect_™_ with laser sensors for reducing limitations of the first [27].

## Brain-Computer Interface

a hardware and software communication system that permits to control computers and external devices through cerebral activity, specifically appropriate for severely disable people [28].

## Surveillance and Tracking

applied to detect ground targets through sensor nodes in a distributed network [29].
